# Inhaled Corticosteroids, Vitamin K Antagonists and Amlodipine Were Associated with an Increased Risk of Acute Periprosthetic Joint Infection in Patients with Total Hip Arthroplasty: A Retrospective Case–Cohort Study

**DOI:** 10.3390/jcm11071842

**Published:** 2022-03-26

**Authors:** Maarten M. Bruin, Ruud L. M. Deijkers, Michaël P. A. Bus, Erika P. M. van Elzakker, Roos Bazuin, Rob G. Nelissen, Bart G. Pijls

**Affiliations:** 1 Department of Orthopedic Surgery, Haga Ziekenhuis, 2545 AA The Hague, The Netherlands; maarten_m_bruin@hotmail.com (M.M.B.); r.deijkers@deijkers.org (R.L.M.D.); r.bazuin@rhoc.nl (R.B.); 2 Department of Orthopaedics, Leiden University Medical Center, 2333 ZA Leiden, The Netherlands; bus.mpa@gmail.com (M.P.A.B.); r.g.h.h.nelissen@lumc.nl (R.G.N.); 3 Department of Medical Microbiology, Haga Ziekenhuis, 2545 AA The Hague, The Netherlands; e.p.m.vanelzakker@amsterdamumc.nl

**Keywords:** periprosthetic joint infection, total hip arthroplasty, total hip replacement, inhaled corticosteroids, vitamin K antagonist, amlodipine, statin, anticoagulants, nifedipine, case–cohort

## Abstract

The perioperative use of certain medication may influence the risk of developing a periprosthetic joint infection (PJI). Inhaled corticosteroids (ICSs) and cardiovascular drugs are widely used against pulmonary and cardiovascular diseases. While oral corticosteroids and anticoagulants have been shown to increase the risk of developing PJI, this is not clear for ICSs. In contrast, some cardiovascular drugs, such as amlodipine, nifedipine and statins, have been documented to show an antimicrobial effect, suggesting a synergistic effect with antibiotics in the treatment of (multi-resistant) microorganisms. We performed a case–cohort study to assess the association between the occurrence of PJI after THA and the use of inhaled corticosteroids, anticoagulants, or previously mentioned cardiovascular agents. In a cohort of 5512 primary THAs, we identified 75 patients with a PJI (1.4%), and randomly selected 302 controls. A weighted Cox proportional hazard regression model was used for the study design and to adjust for potential confounders (age, sex, smoking, and cardiovascular/pulmonary disease). We found ICS use (HR 2.6 [95% CI 1.1–5.9]), vitamin K antagonist use (HR 5.3 [95% CI 2.5–11]), and amlodipine use (HR 3.1 [95% CI 1.4–6.9]) to be associated with an increased risk of developing PJI after THA. The effect remained after correction for the mentioned possible confounders. The underlying diseases for which the medications are prescribed could also play a role in the mentioned association; we believe, however, that the usages of ICSs, vitamin K antagonists and amlodipine appear to be potential modifiable risk factors for PJI, and therefore have to be questioned during preoperative screening and consultation.

## 1. Introduction

Total hip arthroplasty (THA) is a very effective surgical procedure for reducing pain, improving mobility, and improving quality of life in patients with joint disease. In 2010, 332,000 total hip arthroplasties were performed in the United States, and those numbers have been increasing ever since [1]. In approximately 2% of the cases, the implant becomes infected: this is known as periprosthetic joint infection (PJI) [1]. PJI is a major problem after total hip arthroplasty (THA) [1,2,3]. Patients with acutely infected implants often require long and extensive treatment consisting of (multiple) surgical procedures, usually including open debridement without removal of the prosthesis, and antibiotics for several weeks or months (DAIR) [2]. The reported success rate of a DAIR procedure ranges from 31 to 82% [1]. When a DAIR is insufficient, the prosthesis is usually removed and reimplanted either at the time of removal or at a later stage. Eventually, in some cases, final treatment consists of Girdlestone resection arthroplasty, amputation or chronic antibiotic suppression therapy [1]. These treatments have an enormous impact on the patient’s quality of life, as well as an economic impact on society, which is increasing due to rising numbers of multi-resistant microorganisms [4]. In addition, revision surgery in patients with a PJI has a mortality rate which is five times higher at one year after procedure, compared to revision surgery for aseptic failures [5]. Thus, prevention of PJI is vital, both for the patient as well as for society. One important aspect of prevention is preoperative optimization of modifiable risk factors [6]. Medication is one of these important modifiable risk factors. Since this factor can be easily addressed, medication can be substituted for a less harmful alternative or stopped perioperatively.

In particular, immunosuppressive drugs, such as corticosteroids, have been shown to increase the risk for the development of PJIs [7]. However, research has focused on oral use of corticosteroids, whereas corticosteroids are more commonly administered by inhalation, for instance in the treatment of chronic obstructive pulmonary disease (COPD) [8]. With a prevalence of approximately 10% for COPD, a substantial proportion of patients undergoing THA is using inhaled corticosteroids [9]. Although the systemic effects of inhaled corticosteroids on the immune system are less pronounced than that of oral corticosteroids, it is unknown if they are a modifiable risk factor for PJI.

Cardiovascular drugs are widely used for treatment and prevention of cardiovascular diseases. Anticoagulants are of special interest in patients undergoing surgical procedures, as they increase the risk of postoperative (wound) complications such as persistent wound leakage and hematoma formation. These complications are well-known risk factors for the occurrence of PJI [10]. However, the exact effect of the different types of anticoagulants (such as vitamin K antagonists (VKAs), platelet aggregation inhibitors (PAIs)and direct oral anticoagulants (DOACs)) on the risk of PJI remains largely unknown.

In contrast to the aforementioned potentially harmful drugs, in vitro studies suggest that cardiovascular drugs such as amlodipine and nifedipine might have antimicrobial properties, and therefore could be of value as antibiotic-assisting drugs [11]. For example, amlodipine was found to inhibit β-lactamase, and its synergistic effects were positively evaluated in methicillin-resistant *Staphylococcus aureus* [12]. In addition, it has been hypothesized that statins have a beneficial role in the treatment of infections [13].

We therefore set out to answer the following research question: are inhaled corticosteroids, anticoagulants and other cardiovascular drugs (e.g., amlodipine and nifedipine) associated with an increased or with a decreased risk of acute PJI in patients undergoing primary THA?

## 2. Materials and Methods

### 2.1. Patient and Methods

This is a case–cohort study of 5512 patients who underwent primary THA via the direct anterior approach (DAA). All THAs in our study population were performed in the HAGA hospital (a high-volume teaching hospital in the Netherlands) between January 2009 and December 2017. We chose a case–cohort design to allow for efficient assessment of the risk factors, and used the approach of Cai et al. (2004) for sample-size calculations [14]. The study population and the methodology have been previously described [15]. This design provides similar effect estimates and standard errors compared with full cohorts, while at the same time allowing for a high level of detail. In addition, this design is more powerful than nested case–control designs when using a binary predictor [16]. Other studies with similar sample sizes were able to detect risk factors for PJI after joint arthroplasty [17].

### 2.2. Study Cohort and Controls

To minimize variance due to different procedures and surgical approaches, we excluded patients with hemi-arthroplasty, revision surgery and THA through an approach other than the direct anterior approach (DAA). All patients received perioperative prophylactic antibiotics as per local protocol. Low-risk patients received cefazolin and high-risk patients received vancomycin in combination with ciprofloxacin. Patients who used VKAs, PAIs or a DOAC were instructed to temporarily stop using the anticoagulants during the perioperative period, according to hospital protocol. If possible, VKAs were stopped 3–7 days preoperatively, and were re-started at day 2 postoperatively if there were no signs of wound leakage. Patients with VKAs and high thromboembolic risk were bridged according to the 9th ACCP 2012 guidelines [18]. All patients were given low-molecular-weight heparin (prophylactic dosage as dictated by body weight) postoperatively for four weeks or until VKAs or DOACs where adequately restarted.

We randomly selected four controls (for each case) from the study cohort using a random number generator. This resulted in 75 cases and 302 controls. Three cases were also included as controls. This is normal in case–cohort designs, and it indicates that the selection of controls was at random: at baseline (immediately after undergoing THA), a number of patients have not been diagnosed with PJI, but might develop this complication in the future. Hence, when selecting a random sub-cohort of controls at baseline, the percentage of controls who developed PJI should be similar to the incidence of PJI in the overall cohort [19]. The latter was present in our study cohort: 1.4% (72 of 5512) is comparable to 1% (3 of 302).

### 2.3. Cases

All cases developed an acute PJI, defined as a PJI developing within the first 3 months after surgery or an acute hematogenous infection of the prosthesis [1,20]. The diagnosis of PJI was made according to the major and minor Musculoskeletal Infection Society (MSIS) criteria. Major MSIS criteria: 2 or more positive cultures or the presence of a sinus tract. Minor MSIS criteria: elevated C-reactive protein (CRP) and erythrocyte sedimentation rate (ESR), elevated synovial fluid white blood cells (WBC), elevated synovial fluid polymorphonuclear neutrophil percentage (PMN%), presence of purulence in the affected joint, positive histological analyses of periprosthetic tissue or a single positive culture [21]. In the HAGA hospital, acute PJIs were usually treated with an initial DAIR (debridement, antibiotics, and implant retention) procedure.

We consulted with the Dutch Arthroplasty Register (DAR/LROI) to access if revision for infections or DAIRs had been performed in other hospitals for patients in the study cohort [22]. The DAR identified no revisions for infections with exchange of a modular component, nor DAIRs performed in other hospitals, for the patients in our cohort.

### 2.4. Data Collection and Statistical Analyses

Data were extracted retrospectively from the hospital’s electronic information system HiX or paper medical records by the researchers in 2019, and recorded in Castor Electronic Data Capture software. We collected the demographic data, perioperative use of inhaled corticosteroids, anticoagulants (VKAs, PAIs and DOACs), amlodipine use, nifedipine use, statin use and information on potential confounders. During preoperative screening, the anesthesiologists documented the medication-use of our patients. Due to probable small groups, we chose not to analyze the different sorts or brands of the mentioned medicines individually.

PJI is a time-to-event outcome, and the effect of ICSs and the mentioned cardiovascular and pulmonary drugs on PJI risk was analyzed with Kaplan–Meier statistics and weighted Cox proportional hazards regression. A weighted method according to Barlow et al. (1999) was used to calculate the hazard ratios (HR) and their 95% confidence interval (CI) [23]. Sub-cohort controls were weighted by the inverse of the sampling fraction α (=302 controls/5512 entire cohort = 0.055). The cases’ weight outside the sub-cohort was always one at failure. Hence, the following weights were used: 1 for a case outside the sub-cohort at failure, 18 (=1/0.055) for a case in the sub-cohort before failure, 1 for a case in the sub-cohort at failure, 18 for a sub-cohort control. A Kaplan–Meier curve was made to ascertain that the proportional hazard assumption was not violated.

We selected confounders based on the following criteria [24]:

(1) A confounding factor must be an extraneous risk factor for the disease (i.e., PJI).
(2) A confounding factor must be associated with the exposure (i.e., ICSs and cardiovascular drug use) under study in the source population.
(3) A confounding factor must not be affected by the exposure or the disease. In particular, it cannot be an intermediate (mediator) step in the causal path between exposure and the disease.

Regarding criterion 1, demographic factors such as age and sex, smoking, pulmonary and cardiovascular disease are associated with PJI [25,26]. Regarding criterion 2, cardiovascular drugs are indicated in case of treatment or prevention of cardiovascular diseases, and ICSs are used to treat pulmonary diseases such as asthma and COPD [27]. Smoking is a major cause of pulmonary and cardiovascular diseases (and thus the use of ICSs and cardiovascular drugs) [28]. These factors do not oppose criterion 3, so they were considered possible confounders and included in the model. Taken together, the anticoagulants, amlodipine, nifedipine and statins were adjusted for the following possible confounders: age, sex, smoking and cardiovascular disease. ICS use was adjusted for age, sex, smoking and pulmonary disease. Analyses were conducted using SPSS 23.0.0.0. and R package “coxphw”, to allow for calculation of robust standard errors [29].

## 3. Results

In our cohort of 5512 primary THAs, we identified 75 cases of PJIs (1.4%). The causative micro-organisms of PJI were: *S. aureus* (*n* = 32), Coagulase-negative staphylococci (*n* = 23), *P. aeruginosa* (*n* = 8), *E. faecalis* (*n* = 13), *E. faecium* (*n* = 1), *Enterobacteriaceae* (*n* = 23), *Streptococci* (*n* = 6) and *Cornyebacterium* ssp (*n* = 4). In 28 PJIs, polymicrobial cultures were present. The majority of cases (*n* = 73, 97%) were early-onset postoperative PJIs (<3 months after the THA). We identified two cases with an acute hematogenous (6 months and 2 years after primary THA) PJI. The 73 early-onset PJIs were treated with a DAIR within 3 months after THA. The two late cases developed symptoms less than 4 weeks prior to DAIR procedure. Regarding the major and minor MSIS criteria, two or more perioperative cultures were positive in 74 cases. In the remaining case, one positive perioperative culture was found, and it met the minor MSIS criteria. The mean duration between the index procedure and DAIR was 36 days (SD 90). The mean follow-up for the controls was 3.8 years (SD 2.3 years, range 15 to 3361 days) (Table 1).

**Figure 1:**
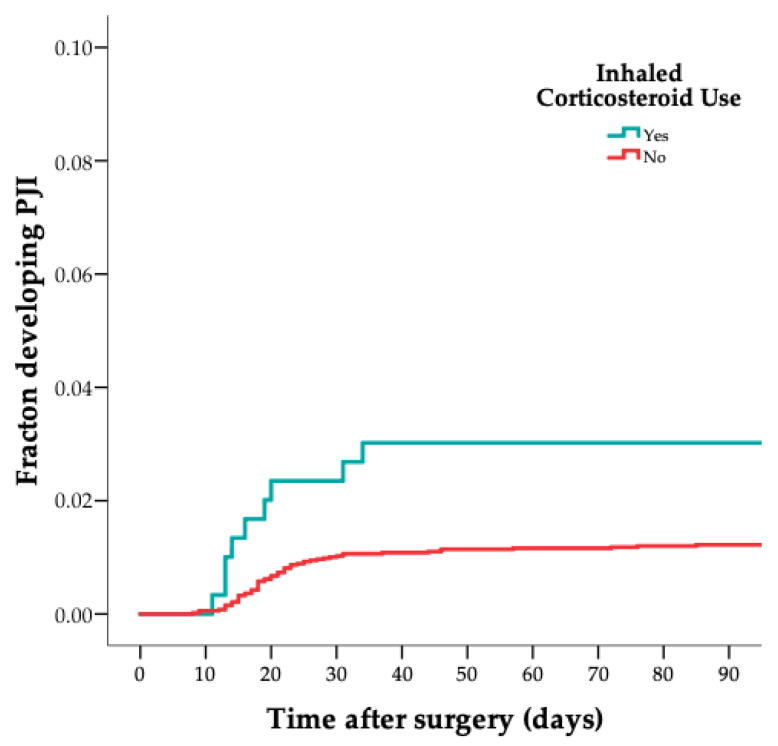
Graph showing risk of PJI according to inhaled corticosteroid use (weighted 1-minus-survival Kaplan–Meier plot). PJI: Periprosthetic joint infection.

**Figure 2:**
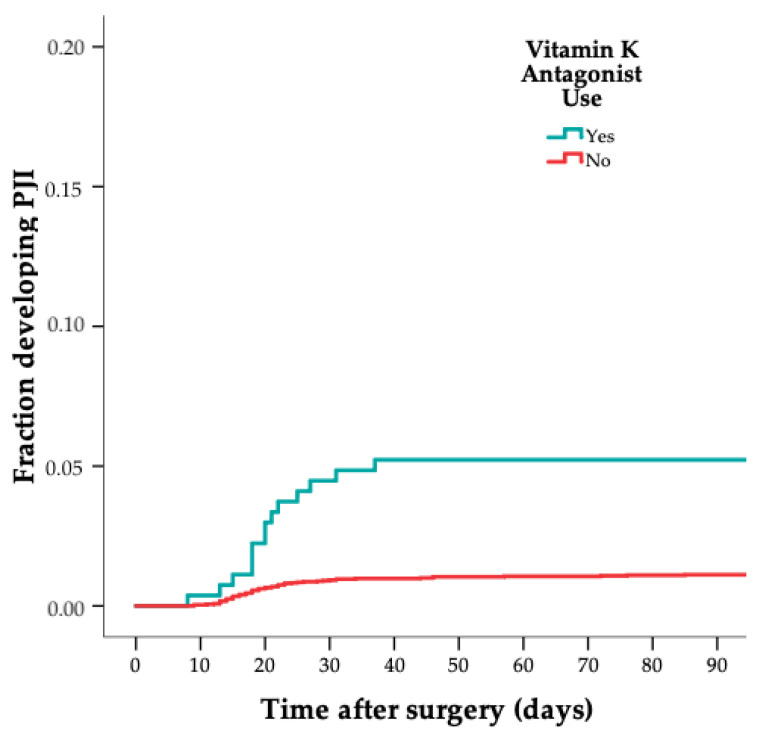
Graph showing risk of PJI according to vitamin K antagonist use (weighted 1-minus-survival Kaplan–Meier plot).

**Figure 3:**
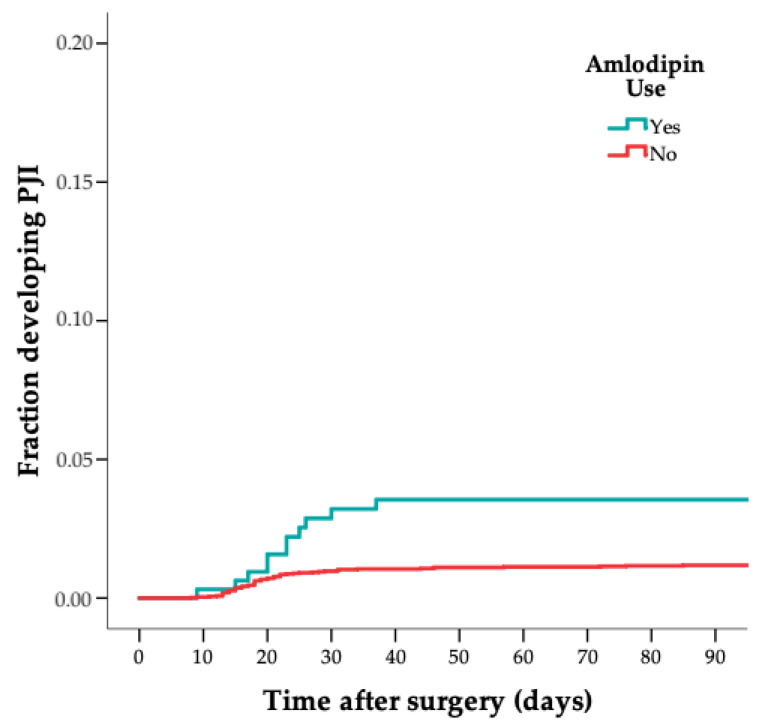
Graph showing risk of PJI according to amlodipine use (weighted 1-minus-survival Kaplan–Meier plot).

Of 75 patients with a PJI, 10 (13%) used ICSs perioperatively, compared to 16 out of 302 in the control group (5%) (crude HR 2.6 [CI 1.1–5.9]); see Table 2 and Figure 1. In addition, 16 out of 75 patients with a PJI (21%) used a VKA perioperatively, compared to 14 (5%) out of 302 in the control group (crude HR 5.3 [CI 2.5–11]); see Table 2 and Figure 2. Lastly, 11 out of 75 cases (15%) used amlodipine perioperatively, compared to 17 out of 302 in the control group (6%) (crude HR 3.1 [CI 1.4–6.9]); see Table 2 and Figure 3. After multivariable adjustment for possible confounders, the increased estimated risk for ICSs, VKAs and amlodipine use on PJI remained; see Table 3.

Of the 75 patients with PJI, 19 patients (25%) used PAIs, versus 58 patients (19%) in the control group (crude HR 1.5 [CI 0.8–2.6]); see Table 2. For statins, 25 patients (33%) with a PJI used statins perioperative versus 75 (25%) in the control group (crude HR 1.5 [CI 0.9–2.6]); see Table 2. Thus, the perioperative use of PAIs and statins was not associated with an increased risk of PJI.

The number of patients who used nifedipine (*n* =1 for cases versus *n* = 4 for controls) and DOACs (*n* = 0 for cases versus *n* = 4 for controls) was very limited. Therefore, we excluded them from further analysis, since these results would not be reliable.

Figure 1, Figure 2 and Figure 3 show the risk of PJI according to ICS, VKA and amlodipine use (weighted 1-minus-survival Kaplan–Meier plot). The two acute hematogenous PJI cases were excluded in a sensitivity analysis, and the results remained similar.

## 4. Discussion

PJIs can be devastating, requiring expensive and prolonged treatment with recurrent hospital admissions and multiple surgical procedures, including debridement, one- or two-stage revision surgery or definitive resection arthroplasty [1,2]. Therefore, we believe that preoperative screening for patients with increased risk, optimizing modifiable risk factors before surgery, and counseling patients is important in decreasing the incidence of this major complication. In this case–cohort study, our aim was to evaluate an association between the use of ICSs, VKAs, PAIs, DOACs, nifedipine, amlodipine and statins, and the risk for developing a PJI. We found an association between the occurrence of PJI and the use of ICSs, VKAs and amlodipine use. This effect remained after correcting for possible confounders (i.e., age, sex, smoking and pulmonary/cardiovascular disease). The incidence of PJI in our cohort of 5512 patients undergoing THA through DAA between 2009 and 2017 was 1.4%. This was within the range of reported infections rates for the DAA from other authors [30,31].

Oral immunosuppressive drugs have previously been described to increase the risk of PJI by suppressing the immune system. For example, Salt et al. (2017) reported an increased risk for oral prednisone use (OR 1.66, 95% CI 1.28–1.97) [7]. We are not aware of other clinical studies describing an association between ICSs and an increased risk for PJI. The systemic effect of inhaled corticosteroids remains partially unclear. Although inhalation corticosteroids seem to be given in low doses, there is a systemic effect that suppresses the immune system, similar to the pathway of the oral corticosteroids. An ICS must be bioavailable to induce a systemic effect in a patient. Lipworth et al. (1999) describe a mechanism of systemic absorption of ICSs [32]. A dose administered by a pressurized metered-dose inhaler is partially deposited in the oropharynx (>60%), and a smaller fraction reaches the lungs (<20%). A small amount is directly absorbed in the oral cavity; another proportion of the oropharyngeal ICS dose is subsequently swallowed and absorbed from the gastrointestinal tract and enters the portal circulation to the liver. There is a varying degree of first-pass metabolism of inactive metabolites in the liver: 70% for beclomethasone, 90% for budesonide and triamcinolone and 99% for fluticasone [32]. The amount of drug that is absorbed from the gastrointestinal tract, and the portion that escapes inactivation by first-pass metabolism in the liver, enter the systemic circulation unchanged. The amount of ICS dose that is delivered to the lungs, executing its desired pharmacological effect, can subsequently partly be absorbed in the systemic circulation [27,32]. For all of the ICSs, except beclomethasone, there is no first-pass transformation in the lungs, meaning that most of the respirable dose absorbed in the lungs is bioavailable in the systemic circulation as unchanged active drug [32]. Most of the absorption and the overall systemic bioavailability is achieved through the pulmonary component, especially in ICSs with a high first-pass metabolism in the liver [32]. Another explanation for the association we found between ICS use and PJI may be the presence of chronic diseases (such as COPD and asthma) in patients, and the effect on the human body. Systemic effects and other diseases are more common in patients with COPD, such as skeletal muscle dysfunction, cardiovascular disease, osteoporosis and diabetes. In addition, COPD is associated with systemic oxidative stress, activation of circulating inflammatory cells and increased plasma levels of pro-inflammatory cytokines [33]. This systemic inflammatory state could be a reason for patients being more susceptible for infections, and could possibly impair the immune response when the body is introduced to micro-organisms. Although we found an even larger effect when adjusted for pulmonary disease, these patients had an almost five times higher risk of having a PJI after primary THA.

We hypothesized that amlodipine would decrease the risk of PJI after THA, based on results from in vitro studies. In contrast to our hypothesis, we found amlodipine to be associated with an increased risk for PJI; this association remained after adjusting for possible confounders. Previously, Kumar et al. (2003) and Yi et al. (2019) found amlodipine to have antibacterial activity, and to inhibit a range of bacteria both in vitro and in vivo (animal models, white mice), and tested its potential as an antimicrobial drug [11,12]. Yi et al. (2019) found that amlodipine exhibited inhibition of a wide range of β-lactamases and synergistic antimicrobial effects in combination with cephalosporines [12]. Kumar et al. (2003) found *Staphylococcus aureus* to be most sensitive towards amlodipine [11]. While the previously described mechanism of amlodipine on β-lactamases has been tested in vitro and in mice models, this effect has not been tested in humans. We are not aware of other clinical studies describing an association between amlodipine and an increased risk of PJI. The mechanism of the increased risk we found is unclear, and we suggest further examination of the underlying mechanism in the future.

Anticoagulants have been described to increase the risk for developing postoperative wound complications, and subsequently, Parvizi et al. (2007) concluded that excessive anticoagulation in the form of low-molecular-weight heparin and warfarin (vitamin K antagonist) was associated with a higher risk for PJIs [10]. Patients with persistent wound drainage are at risk of bacterial ingress into deeper tissue layers, eventually reaching the prosthesis site and colonizing the prosthesis, resulting in a PJI. Additionally, hematomas are good media for bacterial growth, and an infected hematoma may lead to a PJI [1]. In addition, patients with postoperative bleeding and hematoma formation are at greater risk of developing anemia requiring allogenic blood transfusion. This may lead to immunomodulation and an impaired immune response [34]. Restarting VKAs relatively too early after surgery can result in increasing wound complications and hematoma formation. In most patients, VKAs can be stopped perioperatively, and the international normalized ratio (INR) is monitored. Leijtens et al. (2014) describe a high complication rate in patients requiring bridging with LWMH during elective THA [35]. This might explain the association we found between PJI and VKA use. We therefore feel that orthopedic surgeons need to be cautious in balancing the prevention of thromboembolic complications with anticoagulants and the risk of developing a PJI.

This study has several limitations. Firstly, due to the retrospective design, it was not possible to document the bridging status in a reliable way. However, we think patients need to be consulted about the risk of using VKAs, even when patients are not bridged perioperatively. The moment of restarting VKAs and perioperative bridging should be aweighed between the thromboembolic risk and the risk for wound complications and PJI. Secondly, despite our cross-check with the Dutch Arthroplasty Registry (LROI data: DAIR with component exchange), we may have missed acute PJIs because of our large population, although this can be considered non-differential misclassification [22,24]. In most situations, non-differential misclassification of a binary disease will give bias towards the null (no effect) [24]. Thus, if we missed cases (for instance acute hematogenous infections), then our estimates for risk factors are on the conservative side. Therefore, missing cases would not lead to false identification or overestimation of risk factors for acute PJIs. Thirdly, due to the observational design, the observed effect between the use of ICSs, VKAs and amlodipine and the development of PJIs should be interpreted as an association, and further research is necessary to determine possible causality [36]. There is a possibility that the observed association could be confounded by the severity of underlying disease in our patients (ASA score) for which the examined medications are prescribed. In addition, the ASA score in patients with PJI was higher than the ASA score in our sub-cohort. Although the ASA score can be relatively unspecific, other underlying comorbidities could have confounded our results.

Temporarily stopping these medications or switching to less harmful alternatives may reduce the risk of developing PJI. The health risks for temporarily stopping the mentioned medications should be weighed against the risk for PJI, and require prior consultation with the prescribing physician.

## 5. Conclusions

The results of our case–cohort study showed that the use of inhaled corticosteroids, vitamin K antagonists and amlodipine were associated with an increased risk of developing PJI after primary THA. The perioperative use of these medications seems to be a potential modifiable risk factor for PJI, and their use could be re-evaluated during preoperative evaluation for THA.

## Figures and Tables

**Table 1:** Patient demographics.

Variable	Cases (*n* = 75)	Controls (*n* = 302)
Age, mean years ± SD	68.9 ± 10.3	67.7 ± 10.8
Sex *n* (%)		
Female	42 (56)	188 (62)
Male	33 (44)	114 (38)
BMI, mean (SD)	30 (5.4)	27 (4.2)
Obesity (BMI > 30), yes (%) ASA score *n* (%)	28 (38)	57 (19)
ASA 1	7 (9.3)	85 (28)
ASA 2	47 (63)	175 (58)
ASA 3	18 (24)	42 (14)
ASA 4	3 (4.0)	0 (0)
Smoking status, yes *n* (%)	19 (25)	54 (18)
Inhalation corticosteroid use *n* (%)	10 (13)	16 (5)
COPD	3	5
Asthma	4	7
Other	3	4
Anticoagulant use *n* (%)	35 (47)	76 (25)
VKA	16 (21)	14 (5)
PAI	19 (25)	58 (19)
DOAC	0 (0)	4 (1)
Amlodipine use *n* (%)	11 (15)	17 (6)
Nifedipine use *n* (%)	1 (1)	4 (1)
Statin use *n* (%)	25 (33)	75 (25)
Cardiovascular disease status, yes *n* (%)	28 (37)	69 (23)
Pulmonary disease status, yes *n* (%)	8 (11)	24 (8)

BMI: Body Mass Index; ASA: American Society of Anesthesiologists; COPD: Chronic Obstructive Pulmonary Disease; VKA: Vitamin K Antagonist; DOAC: Direct Oral Anticoagulant; PAI: Platelet Aggregation Inhibitor.

**Table 2:** Univariable weighted Cox proportion hazard regression model.

Risk Factor	HR	95% CI
Age, year	1.0	1.0–1.0
Sex (male)	0.8	0.5–1.1
ASA score 3 and 4	2.4	1.4–3.9
Smoking status, yes	1.6	0.9–2.6
Pulmonary disease status, yes	1.4	0.7–2.9
Cardiovascular disease status, yes	1.9	1.2–3.1
Inhalation corticosteroid use	2.6	1.1–5.9
VKA	5.3	2.5–11
PAI	1.5	0.8–2.6
DOAC	NA	NA
Amlodipine	3.1	1.4–6.9
Nifedipine	1.0	0.2–7.2
Statin	1.5	0.9–2.6

ASA: American Society of Anesthesiologists; HR: Hazard Ratio; CI: Confidence Interval; VKA: Vitamin K Antagonist; DOAC: Direct Oral Anticoagulant; PAI: Platelet Aggregation Inhibitor.

**Table 3:** Multivariable weighted Cox proportion hazard regression model for PPI.

	ICS		VKA		PAI		Amlodipine		Statin	
	HR	95% CI	HR	95% CI	HR	95% CI	HR	95% CI	HR	95% CI
Crude *	2.6	1.1–5.9	5.3	2.5–11	1.5	0.8–2.6	3.1	1.4–6.9	1.5	0.9–2.6
Model 1	2.7	1.2–6.1	5.2	2.4–11	1.4	0.8–2.6	3.1	1.4–6.8	1.5	0.9–2.6
Model 2	2.6	1.1–6.2	5.2	2.3–12	1.4	0.7–2.7	3.1	1.3–7.1	1.5	0.8–2.7
Model 3	2.5	1.0–6.1	5.7	2.4–13	1.4	0.7–2.8	3.4	1.4–8.2	1.5	0.8–2.8
Model 4	-		4.8	1.9–12	1.1	0.5–2.2	3.1	1.3–7.3	1.3	0.7–2.4
Model 5	4.5	0.7–30	-		-		-		-	

ICS: Inhaled Corticosteroid; VKA: Vitamin K Antagonist; DOAC: Direct Oral Anticoagulant; PAI: Platelet Aggregation Inhibitor; HR: Hazard Ratio; CI: Confidence Interval; Model 1: Crude HR adjusted for age; Model 2: Crude HR adjusted for sex; Model 3: Crude HR adjusted for smoking; Model 4: Crude HR adjusted for cardiovascular disease; Model 5: Crude HR adjusted for pulmonary disease; * Crude = HR from univariable model (Table 2).

## Data Availability

The data are available upon reasonable request by contacting the corresponding author.
